# Antibiofilm Activity of a Novel Calcium Phosphate Cement Doped with Two Antibiotics

**DOI:** 10.3390/jfb16090320

**Published:** 2025-08-31

**Authors:** Eneko Elezgaray, Cassandra Pouget, Fanny Salmeron, Catherine Flacard, Jean-Philippe Lavigne, Vincent Cavaillès, Mikhael Bechelany

**Affiliations:** 1 Institut Européen des Membranes, IEM, UMR 5635, University of Montpellier, Ecole Nationale Supérieure de Chimie de Montpellier, ENSCM, Centre National de la Recherche Scientifique (CNRS), 34095 Montpellier, France; eneko.elezgaray@umontpellier.fr; 2 IRCM, Institut de Recherche en Cancérologie de Montpellier, INSERM U1194, Université Montpellier, 34000 Montpellier, France; fanny.salmeron@inserm.fr; 3 NORAKER SAS, 60 Av. Rockefeller, 69008 Lyon, France; c.flacard@noraker.com; 4 VBIC, INSERM U1047, University of Montpellier, Service de Microbiologie et Hygiène Hospitalière, CHU Nîmes, 30029 Nîmes, France; cassandra.pouget@inserm.fr (C.P.); jean-philippe.lavigne@umontpellier.fr (J.-P.L.)

**Keywords:** calcium phosphate cement, antibiotics, antibacterial activity, biofilm, osteomyelitis

## Abstract

This study presents the development of a degradable and biocompatible calcium phosphate cement (CPC) co-loaded with gentamicin (1.25 wt%) and vancomycin (4.25 wt%) for the local treatment of polymicrobial bone infections. The antibiotics were incorporated—individually or in combination—into the solid phase of Graftys^®^ Quickset (GQS), an injectable CPC. Antibiotic loading modifies some of the intrinsic properties of the GQS cement. Porosity exceeded 53%, compressive strength reduced around 5 MPa, which is comparable to calcium sulphates cements, and the setting time, although extended, remained within the clinically acceptable threshold (<20 min), ensuring suitable handling. A burst release of both antibiotics was observed within the first 24 h, with sustained release over time and no cytotoxic effects on human osteoblasts. The dual-loaded cement exhibited broad-spectrum antibacterial activity against both Gram-positive and Gram-negative strains, including methicillin-resistant isolates, in both planktonic and biofilm forms. Notably, the combination of both antibiotics demonstrated superior efficacy compared to either antibiotic alone. These findings suggest that this dual-antibiotic-loaded CPC offers a promising strategy for localised treatment of complex bone infections such as osteomyelitis, where polymicrobial involvement and antibiotic resistance pose significant therapeutic challenges.

## 1. Introduction

Osteomyelitis is a serious and increasingly common complication in orthopaedic surgery, predominantly caused by bacterial infections. Osteomyelitis can develop through several distinct pathways: direct inoculation of pathogens during trauma (e.g., open fractures or prosthesis implantation; this type of infection can impact patients of all ages and any bone), contiguous spread from adjacent infected tissues (as observed in patients with diabetic foot ulcers or pressure sores), or hematogenous dissemination, particularly in children and the elderly, where circulating bacteria colonise damaged or weakened bone tissue. A critical distinction exists between acute and chronic osteomyelitis. Chronic osteomyelitis is typically associated with low-grade, persistent infections that often lead to the formation of necrotic bone tissue (sequestrum) [1,2].

Osteomyelitis is most frequently caused by Gram-positive bacteria, especially *Staphylococcus aureus* (SA) and *Staphylococcus epidermidis* (SE), which together have accounted for approximately 80% of cases in Europe over the past two decades. However, the incidence of Gram-negative infections has risen significantly, from 25% to nearly 35%. Multidrug-resistant (MDR) Gram-positive pathogens, including methicillin-resistant *S. aureus* (MRSA), represent 7.6% of cases. A similar trend has been observed for MDR Gram-negative bacilli such as *Pseudomonas aeruginosa* (PA), whose prevalence has increased from 9.3% to 15.8%. Other pathogens, including enterococci and streptococci, are less frequently reported [3,4].

Effective osteomyelitis management requires a personalised approach combining systemic antibiotics and surgical intervention tailored to the site of infection, microbial profile, and the host’s immune status. However, systemic antibiotics often fail to achieve therapeutic concentrations at the bone infection site due to poor bone vascularisation and the protective nature of bacterial biofilms [5,6]. Consequently, surgical debridement of the necrotic part of the bone coupled with local antibiotic delivery targeting the identified pathogens is crucial to reduce the risk of treatment failure [1,7,8].

Calcium phosphate cements (CPC) are widely used as bone fillers due to their biocompatibility and chemical similarity to the mineral phase of bone. They offer advantages such as mouldability, injectability, and in vivo self-setting properties, facilitating minimally invasive surgical procedures. CPCs also exhibit mechanical strength comparable to cancellous bone, degrade more slowly than calcium sulphate (CS) [9], and unlike polymethyl methacrylate (PMMA) do not undergo exothermic setting reactions, thereby avoiding thermal and chemical cytotoxicity [10,11]. Incorporating antibiotics into bone cements provides a promising strategy to achieve high local drug concentrations at the infection site [12,13,14,15].

Various strategies for incorporating drugs into CPCs have been explored, including mixing the drug with the solid phase, dissolving it in the liquid phase, immersing the cement in drug solutions, or embedding drug-loaded microspheres [13,16]. Most studies have focused on loading CPCs with a single antibiotic, often using different carriers [17,18,19,20,21,22,23]. Additional research has explored antibiotic incorporation into PMMA [24,25,26,27] and CS [28,29,30]. To our knowledge, no prior study has reported the co-loading of two antibiotics into a CPC matrix.

In the present work, we aimed to develop a composite CPC formulation with properties intermediate between PMMA and CS, capable of locally releasing both gentamicin and vancomycin to prevent and/or treat bone infections. Gentamicin, an aminoglycoside, is commonly used against aerobic Gram-negative bacteria [31,32,33], while vancomycin, a glycopeptide that inhibits bacterial cell wall synthesis, is recommended for Gram-positive infections, including methicillin-resistant staphylococci [34,35,36,37]. Both antibiotics were incorporated, individually and in combination, into the solid phase of the Graftys^®^ Quickset (GQS), a clinically used injectable CPC [38]. We then examined the impact of their incorporation on the cement’s properties and assessed in vitro cytocompatibility and antibacterial activity against multiple bacterial strains in both planktonic and biofilm forms.

## 2. Materials and Methods

### 2.1. Materials

The injectable cement powder used in this study was Graftys^®^ Quickset (GQS) commercialised by Graftys SAS (Aix-en-Provence, France) [39]. The di-sodium hydrogen phosphate anhydrous (Na_2_HPO_4_, ≥99.0%, CAS 7558-79-4) used in the hardening solution was purchased from VWR (Rosny-sous-bois, France). The antibiotics used were gentamicin sulphate (G) from Merck (Darmstadt, Germany) (CAS 1405-41-0) and vancomycin hydrochloride (V) from Apollo Scientific (Manchester, UK) (CAS 1404-93-9). The bacterial strains included *S. aureus* ATCC 6538 (SA), MRSA reference strain USA300 (MRSA), and *P. aeruginosa* ATCC 9027 (PA). *S. epidermidis* (SE) and a methicillin-resistant *S. epidermidis* strain (MRSE) were clinical isolates obtained from osteoarticular infections and provided by the Department of Microbiology, CHU of Nîmes [40,41]. Reagents for cytocompatibility assays included AlamarBlue™ HS Cell Viability Reagent (Cat N°: 150100) and spectrophotometric-grade Ninhydrin (CAS: 485-47-2), both from ThermoFisher (Breda, The Netherlands). Cell culture reagents included DMEM/F-12 (Gibco 21041-025), dimethyl sulfoxide (DMSO; BDH Prolabo 23,486.297), 10% foetal bovine serum (FBS; Eurobio CVFSVF00-01), 1% penicillin/streptomycin (Gibco 15140-122), and 0.05% trypsin-EDTA (Gibco 25300-054). The MTT reagent (3-(4,5- dimethylthiazol-2-yl)-2,5-diphenyl tetrazolium bromide; 98%, CAS 298-93-1) was purchased from Sigma–Aldrich (Saint-Louis, MO, USA).

### 2.2. Cement Preparation

Cements were prepared by mixing 5.95 g of GQS powder and 2.7 mL of osmosed water containing 0.5% (*w*/*w*) Na_2_HPO_4_. Gentamicin and vancomycin were incorporated into the solid phase either individually or in combination. After mixing using a mortar for 1 min, the powder was transferred into a Medmix (Baar, Switzerland) P system syringe. Antibiotic concentrations were 1.25% (*w*/*w*) for gentamicin and 4.25% (*w*/*w*) for vancomycin, with the same concentrations used in the dual-loaded formulation. After 2 min of mixing in the syringe, the cements were injected into moulds to form discs (6 mm diameter and 2 mm thickness). After setting for 30 ± 2 min, the moulds were dried in an oven at 50 °C for 24 h. After the treatment, the pellets were then removed and sterilised under UV light (405 nm) for 1 h.

### 2.3. Mechanical Properties

The mechanical properties of the cements (both measurement of compressive and flexural strengths) were assessed using a dynamic mechanical analysis system (Metravib (Limonest, France) 50N) in controlled force mode. Cylindrical samples were prepared in Teflon moulds and immersed in 0.9% (*w*/*w*) saline solution for 24 h. The measurements were carried out in two ways: either directly after removing the samples from the solution, or after drying them for 24 h at 50 °C. For the compressive strength measurements, the size of the cylinders was 12 mm in length and 6 mm in diameter. For the flexural strength analysis, the size was 20 × 4 × 4 mm. The samples were compressed (at 0.01 mm/s for mechanical strength and 0.06 mm/min for bending) until they broke. The stress at which the sample began to break was measured. At least five replicates were used for each condition.

### 2.4. Scanning Electron Microscopy

The surface morphology of the set cements was imaged using a Hitachi S4800 scanning electron microscope (Hitachi, Tokyo, Japan).

### 2.5. Porosity

Porosity was evaluated using the same cylindrical samples prepared for mechanical testing. After drying, the mass (*m*) and dimensions (*D* = diameter, *l* = length) of each sample were measured using a caliper. The following formula was used to determine the density ($\rho_{sample}$) of each cylinder:

$$m/l\times \pi \times {\left(D/2\right)}^{2}=\;\rho_{sample}$$

The theoretical density provided by the manufacturer was $\rho_{theo}$ = 2.77 g·cm^−3^. Total porosity was then calculated as:

$$\left(1-\left(\rho_{sample}/\rho_{theo}\right)\right)\times 100=P_{tot}(\%)$$

### 2.6. Injectability

Injectability was measured by weighing the empty syringe (*W_0_*), then weighing it again after adding the solid and liquid phases (*W_1_*) corresponding to the volume of a commercial dose (6 cc). After 2 min of mixing, the cement was injected, always by the same operator, and the syringe with residual cement was weighed (*W_2_*). The diameter of the syringe nozzle through which the cement was injected is 2 mm. Injectability was then calculated as:

$$\left(W_{2}-W_{0}\right)/\left(W_{1}-W_{0}\right)\times 100=injectability\;(\%)$$

### 2.7. X-Ray Diffraction (XRD)

XRD analysis was performed using a Bruker D8 Discover Theta-Theta diffractometer (Brucker, Billerica, MA, USA) equipped with a copper anode tube operating at 40 kV and 40 mA and fitted with a front monochromator for obtaining monochromatic incident radiation with a wavelength equal to Cu-Ka1 radiation (1.5405929 Å), a spinner-type sample holder, and a high-energy resolution linear detector. Powder for this analysis was obtained by crushing cylinders synthesised by the method described before with an agate mortar.

### 2.8. Setting Time and Cohesiveness

The initial setting time (IT) was measured using a weighted Gillmore needle sized in accordance with ASTM C 266-04 [42]. The needle used for IT determination had a diameter of 2.12 mm and a mass of 113 g.

### 2.9. Cytocompatibility

Human foetal osteoblasts (hFOB, ATCC CRL-11372) were stimulated with 100 µL of each dilution from an initial elution of each of the pellets dosed at 0.2 g/mL (CPC, CPC + G, CPC + V, CPC G + V). Eluates were collected for each condition after 24 h of incubation using DMEM F:12 phenols red free (complete). After 24 h, cell viability was analysed using the MTT assay. Cells were incubated with 100 µL of culture medium containing 0.05 mg·mL^−1^ MTT for 3 h. The resulting purple-coloured formazan crystals, due to MTT reduction by living cells, were solubilised by the addition of 100 µL of DMSO, and absorbance was measured at 560 nm using a Multiskan microplate spectrophotometer (ThermoFisher). Untreated hFOB cells served as a control.

### 2.10. Release Kinetics

Pellets of each cement (1 g) were immersed in 5 mL of phosphate buffer saline (PBS) and incubated at 37 °C. Samples were prepared in triplicate. The experiment was conducted over a period of seven weeks. During the first week, 5 mL of PBS was collected and replaced with 5 mL of fresh PBS every 24 ± 2 h. Samples were stored at 4 °C until analysis.

Additionally, a paste of freshly mixed cements (6 cc) was immersed in triplicates in 25 mL of PBS and incubated at 37 °C, following a modified protocol based on Stravinskas et al. [43]. Following the protocol, at each time point, 20% of the PBS was removed and replaced by fresh PBS daily during 7 days. Thereafter, once a week before collection, part of the solution was exchanged using the following formula:

$$Liquid\;to\;replace=25\times \left(\;1-0.8^{n}\right)\;mL$$

where *n* is the number of times the liquid should have been replaced if it was still replaced daily. Then, the sample collection was made as before.

Vancomycin concentrations in the samples were quantified by UV absorbance at a wavelength of 281 nm using a UVmc2 spectrophotometer (SAFAS, Monaco, Monaco).

Gentamicin concentrations were determined by mixing samples (1:1) with a ninhydrin solution previously dissolved in osmosed water (5 mg/mL), heating at 95 °C for 15 min, and centrifuging at 9000 rpm for 1 min, following the method of Frutos et al. [44]. Absorbance was measured at 402 nm within 30 min using the same spectrophotometer.

### 2.11. Antibacterial Activity

Eluates from cement pellets were prepared by incubating 0.4 g of each cement condition (CPC, CPC + G, CPC + V, CPC G + V) in 1 mL of Lysogenic Broth (LB) at 37 °C for 24 h. After incubation, eluates were diluted with LB to reach a final volume of 2 mL (e.g., for 1:2 dilution= 1 mL of initial eluate of CPC + 1 mL of LB). Overnight bacterial pre-cultures grown in LB were diluted to an OD_600_ of 0.113 for SA, MRSA, and PA and 0.1 for SE and MRSE, corresponding to final bacterial concentrations of 10^8^ CFU/mL for SA, MRSA, and PA and 10^7^ CFU/mL for SE and MRSE. These suspensions were further diluted in LB: 1:50 for SA, MRSA, and PA; 1:10 for SE; and 1:5 for MRSE. For each condition, 2 mL of the diluted bacterial suspension was added to the respective eluate. A positive control consisted of 5 mL of bacterial suspension mixed with an equal volume of LB.

After 4 h of incubation at 37 °C in a rotary incubator, 100 µL of each condition was transferred to a 96-well plate (*n* = 6 replicates per condition). Antibacterial activity of the antibiotics alone or in combination was assessed using the resazurin microtiter assay (REMA): 11 µL of resazurin solution was added to each well, followed by 1 h of incubation at 37 °C. Resazurin reduction by metabolically active bacteria resulted in a pink fluorescent signal, which was measured at 590 nm using a Multiskan™ microplate spectrophotometer (ThermoFisher, Breda, Netherlands). For PA, which interfered with the fluorescence-based REMA assay, antibacterial activity was assessed by measuring OD_600_ after 4 h of incubation at 37 °C in a rotary incubator, using the same microplate reader.

### 2.12. Biofilm Formation Under Microfluidic Conditions

Biofilm formation was studied using the BioFlux™ 200 microfluidic system (Fluxion Bioscience Inc., Alameda, CA, USA). Cement pellets were placed in the flow channels. Bacterial suspensions were prepared from overnight cultures in brain heart infusion (BHI) broth, and adjusted to OD_600_ = 0.1 ± 0.05. The microfluidic channels were initially primed with 500 µL of BHI at 1 dyne/cm^2^ for 10 min, then inoculated with the bacterial suspension at the same shear stress for 30 min. Cement pellets were positioned inside the channels and the system was maintained at 37 °C on a heated platform. Biofilms were promoted by continuously flowing fresh bacterial suspension through the channels for 48 h at a flow rate of 0.2 dyne/cm^2^ and 37 °C. At the end of the incubation, the biofilms were imaged in situ using an inverted microscope (DM IRB, Leica Biosystems, Nanterre, France) equipped with a CoolSNAP FX camera (Roper Scientific, Lisses, France) and a 40× objective (Leica Biosystems, Nanterre, France). To ensure reproducibility, predefined reference points within the channels were used for consistent imaging across samples. Images were acquired using MetaVue™ v1.0 software (Molecular Devices, Sunnyvale, CA, USA). Biofilm analysis, including scale bar addition and biofilm quantification, was performed using ImageJ^®^ v1.54p. Biofilm biomass was segmented by applying the software’s automatic threshold function, using the default settings to minimise subjective bias. Images were converted to 16-bit grayscale and thresholded automatically to distinguish foreground biomass from background interstitial space. Finally, the ‘Analyze Particles’ function of ImageJ^®^ was used to quantify the percentage of channel area covered by biofilm.

### 2.13. Statistical Analysis

Results are presented as mean ± standard deviation (SD). Statistics were performed using GraphPad Prism (Version 8.0.1). Depending on the dataset, comparisons were made using *t*-tests, one-way ANOVA, or two-way ANOVA. For one-way ANOVA, *p*-values were adjusted using the Brown–Forsythe and Welch multiple comparison test. Statistical significance was indicated as follows: ns *p* > 0.32; * *p* < 0.05; ** *p* < 0.01; *** *p* < 0.001.

## 3. Results

### 3.1. Performance Characteristics of the Cements

Gentamicin and vancomycin were incorporated into the solid phase of GQS cement, either individually or in combination, resulting in four different cements to be tested: CPC (control), CPC + G, CPC + V, CPC + G + V. Antibiotic concentrations were selected based on existing commercial formulations and EUCAST recommendations [45,46,47]: 1.25% (*w*/*w*) for gentamicin and 4.25% (*w*/*w*) for vancomycin.

**Figure 1:**
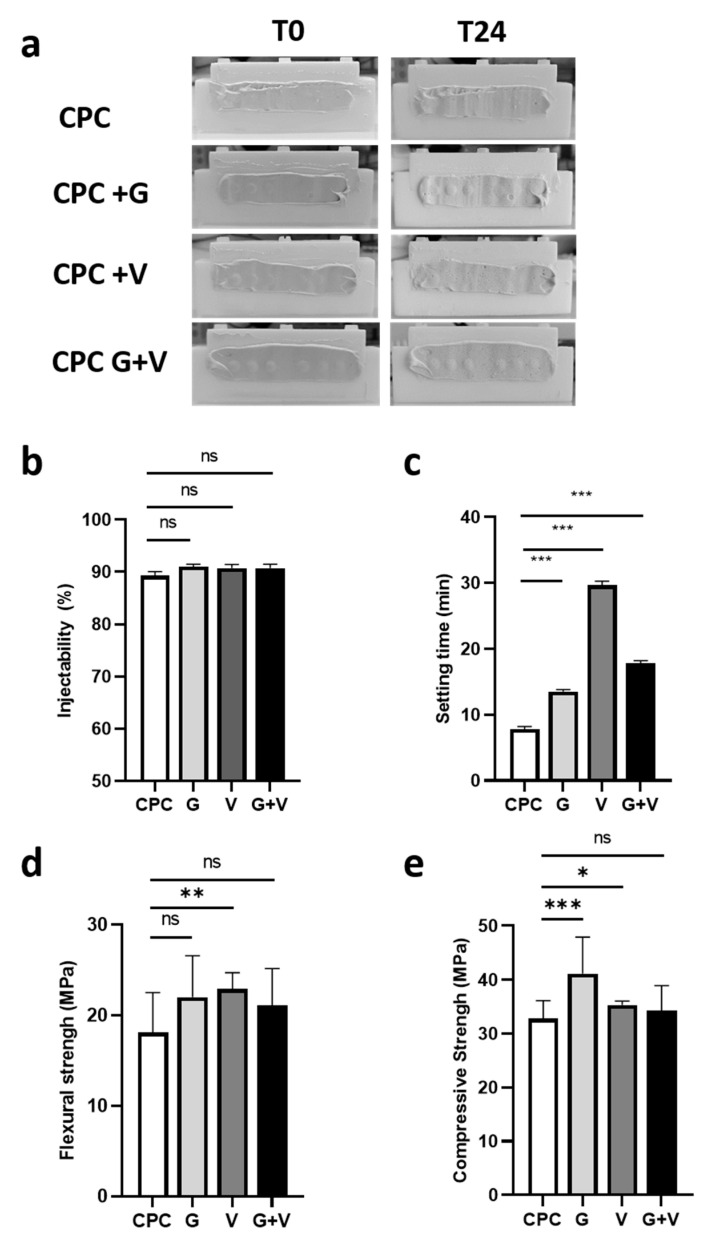
Effect of gentamicin and/or vancomycin on cement characteristics. (**a**) Photographs of the cement coatings before and after 24 h immersion in the saline solution at 37 °C; (**b**) Injectability assays performed in triplicates; (**c**) Initial setting time (± 15 s) measured in triplicate using a Gillmore needle; (**d**) Flexural and (**e**) compressive strength measured on 6 samples per conditions. CPC refers to the unloaded control cement. Data are presented as mean ± standard deviation (SD). Statistical significance: ns *p* > 0.32; * *p* < 0.05; ** *p* < 0.01; *** *p* < 0.001.

Following mixing, the different cements were injected into moulds (Figure 1a). After 24 h of immersion in saline solution, the coatings remained unchanged, indicating that the antibiotic incorporation had no effect on cement cohesiveness. Injectability, a critical parameter for clinical usability, was unaffected by antibiotic loading. As shown in Figure 1b, all formulations exhibited injectability rates around 90%.

To minimise surgical time and potential complications, the setting time of a cement must remain within acceptable limits. As shown in Figure 1c, gentamicin significantly prolonged the setting time from 7 min 50 s (CPC) to 13 min 30 s (CPC + G). Vancomycin had a more pronounced effect, increasing the setting time to 29 min 40 s (CPC + V). Interestingly, the dual-loaded formulation (CPC + G + V) exhibited an intermediate setting time of 17 min 50 s.

Compressive strength was enhanced by antibiotic incorporation (Figure 1e). CPC + G showed the highest strength (41.14 MPa), followed by CPC + V (35.33 MPa), while CPC + G + V remained comparable to the control (34.24 vs. 32.86 MPa, respectively). Flexural strength also improved slightly (Figure 1d), with CPC + V reaching 22.95 MPa compared to 18.10 for CPC. However, the increases for CPC + G and CPC + G + V were not statistically significant compared to the control, (22.01 and 21.09 MPa, respectively).

When analysed under wet conditions, there is a significant drop in both the flexural and compressive strengths of the samples (Supplementary Figure S3a,b). Cement alone has a compressive strength of 14 MPa, followed by CPC + V, which remains above 10 MPa. Adding gentamicin alone reduces the mechanical strength by half (to 6.5 MPa), and adding the two antibiotics together reduces it even further (to 4.5 MPa). The same trend was observed in the assessment of flexural strength, with values of around 4 MPa for cement alone, around 2 MPa for the two conditions with antibiotics alone, and 0.45 MPa for CPC G + V.

### 3.2. Structural Properties

**Figure 2:**
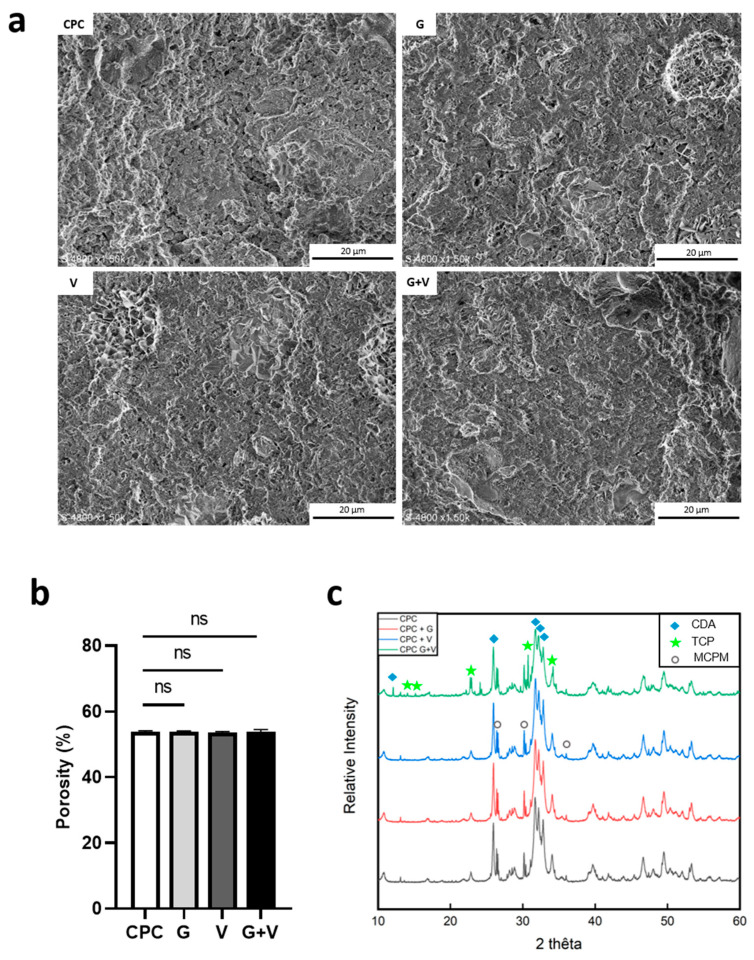
Structural properties of the CPC loaded or not with gentamicin (G) and/or vancomycin (V). (**a**) SEM photographs of the internal surface of cement cylinder cut in half after 24 h immersion in saline at 37 °C and drying at 50 °C; (**b**) Porosity measured on 6 cylinders per condition, expressed as mean ± standard deviation (SD). Statistical significance: ns *p* > 0.32; (**c**) XRD diffractograms of powered cement samples (CPC, CPC + G, CPC + V, CPC + G + V). CPC represents the unloaded control.

To assess the macrostructure, cylindrical blocks of each cement were generated as described in the experimental section. Figure 2a shows the inside surface of the cylindrical blocks corresponding to the four cements after cutting them in half. All formulations exhibited a homogeneous internal structure. Visible pores in CPC + G and CPC + V may reflect air entrapment or voids left by dissolved antibiotics in the liquid phase during hardening. Needle-like structures, indicating calcium-deficient apatite crystal formation, were observed within these pores. Porosity, an important parameter for cell proliferation, was also quantified. As shown in Figure 2b, antibiotic addition had no effect on cement porosity, with porosity values ranging from 53.6% (CPC + V) to 53.9% (CPC). CPC + G + V exhibited a porosity of 53.8%, nearly identical to the control. A mercury porosity experiment was carried out to study the pore size distribution with the addition of antibiotics (Table S2). The overall porosity was around 47% with this technique on CPC. A drop in porosity was observed when gentamicin was added (45.8%), and an even greater drop was observed when both antibiotics were added (43.6%). CPC + V porosity remained stable at around 47%. Decreases in the average pore size were also observed when gentamicin and both antibiotics were added, with an average pore size of 16 nm compared to 25 nm for CPC alone. Finally, XRD analysis was performed to evaluate the crystalline phases. Diffractograms for CPC + G and CPC + V were similar to CPC (Figure 2c). The characteristic peaks for alpha-TCP and CDHA (JCPDS 09-348 and 09-432, respectively) can be seen in the diffractogram of CPC + G + V, indicating that the reaction was not complete. The intensity of the peaks, which are characteristic of the CDA, is also reduced, suggesting a reduction in particle size.

### 3.3. Antibiotics Release and Cytocompatibility

**Figure 3:**
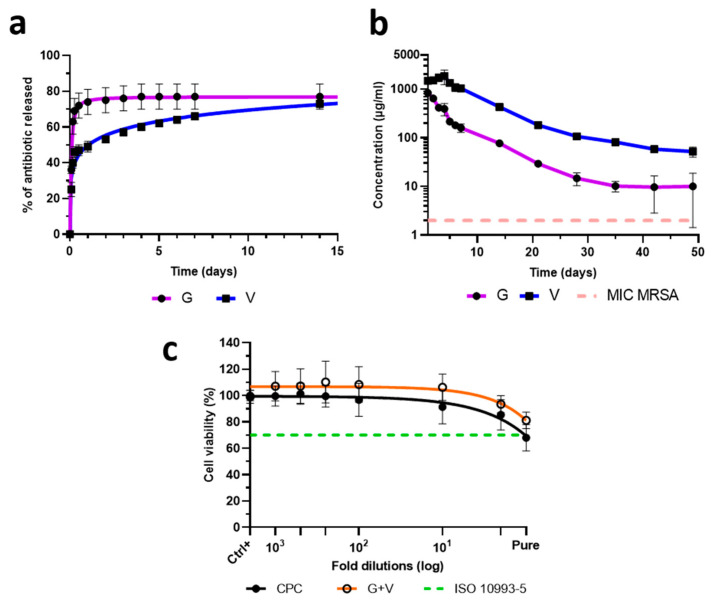
Antibiotic release kinetics and cytocompatibility of unloaded CPC and the CPC loaded with gentamicin and/or vancomycin. (**a**) Cumulative release (%) of gentamicin and vancomycin from CPC + G + V in PBS at 37 °C at different time points; (**b**) Local concentration (µg/mL) of gentamicin and vancomycin in PBS at 37 °C measured over 49 days. Assays were made in triplicates from CPC + G + V samples. 20% of the volume was collected every day and immediately stored at 4 °C until measurement. Results are expressed as mean ± standard deviation (SD); (**c**) Cytotoxicity of 24 h eluates on hFOB cells assessed by MTT assay. All measurements were performed in six replicates.

The release kinetics of gentamicin and vancomycin differed markedly (Figure 3a). Gentamicin exhibited a high release (75%) within the first 24 h, while vancomycin showed a more gradual release (45% on day one and 73% on day 14).

The freshly mixed cements resulted in very high initial antibiotic concentrations in eluates: 830 µg/mL for gentamicin and 1460 µg/mL for vancomycin. After 49 days, the vancomycin concentration remained above 50 µg/mL, while the gentamicin concentration was around 10 µg/mL (Figure 3b), even if almost all of the gentamicin was released in the first few hours.

In order to verify that the antibiotic concentrations achieved upon the burst phase did not have a toxic effect on cells present at the implantation site, cytotoxicity tests were performed using hFOB osteoblasts (Figure 3c). The control cement (CPC) exhibited mild cytotoxicity, but no additional toxicity was observed with antibiotic-loaded formulations. Notably, CPC + G + V appeared to slightly reduce cytotoxicity compared to CPC alone. These results suggest that the burst-phase antibiotic concentration do not adversely affect osteoblast viability.

### 3.4. Antibacterial Activity on Agar Plates

To evaluate the antibacterial activity of the different cements, five representative bacterial strains were selected, including *S. aureus* (SA), *S. epidermidis* (SE)—both methicillin-sensitive and methicillin-resistant (MRSA, MRSE), and *P. aeruginosa* (PA).

**Figure 4:**
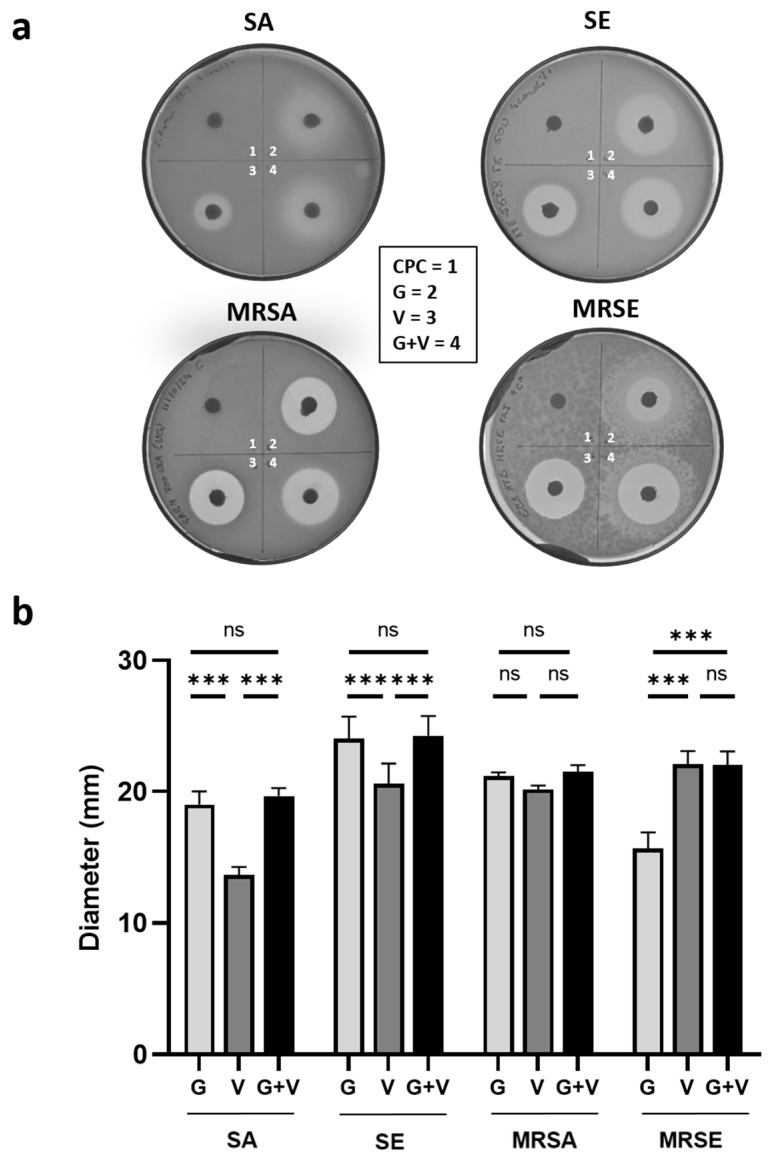
Antibacterial activity of CPC doped with gentamicin and/or vancomycin on agar plates. (**a**) Representative images of inhibition zones of the antibiotic-loaded CPC ((1) CPC unloaded control, (2) CPC + G, (3) CPC + V, (4) CPC + G + V) after 24 h incubation at 37 °C with SA, SE, MRSA, and MRSE. Assays were performed in triplicates. (**b**) Quantification of inhibition zone diameters (mean of three measurements per plate, *n* = 3) ex pressed as mean ± standard deviation (SD). Statistical significance: ns *p* > 0.32; *** *p* < 0.001.

Agar diffusion assays were performed to evaluate the inhibitory effects of the different cements. Representative images of the inhibition zones after 24 h of incubation at 37 °C are shown in Figure 4a, with the corresponding zones’ diameters presented in Figure 4b. As expected, the control cement (CPC) showed no antibacterial effect. Gentamicin exhibited stronger activity than vancomycin against SA and SE. In contrast, vancomycin was more effective against MRSE, while both antibiotics showed similar efficacy against MRSA. Notably, the combination of both antibiotics (CPC + G + V) was equal to or more effective than the most active single antibiotic, particularly against PA, where the combination matched the effect of gentamicin alone, while, as expected, vancomycin alone had no measurable activity (Figure S1a,b).

### 3.5. Antibacterial Activity in Liquid Cultures

To further validate the antibacterial effects of the cements under more physiologically relevant conditions, bacterial viability was assessed in liquid cultures.

**Figure 5:**
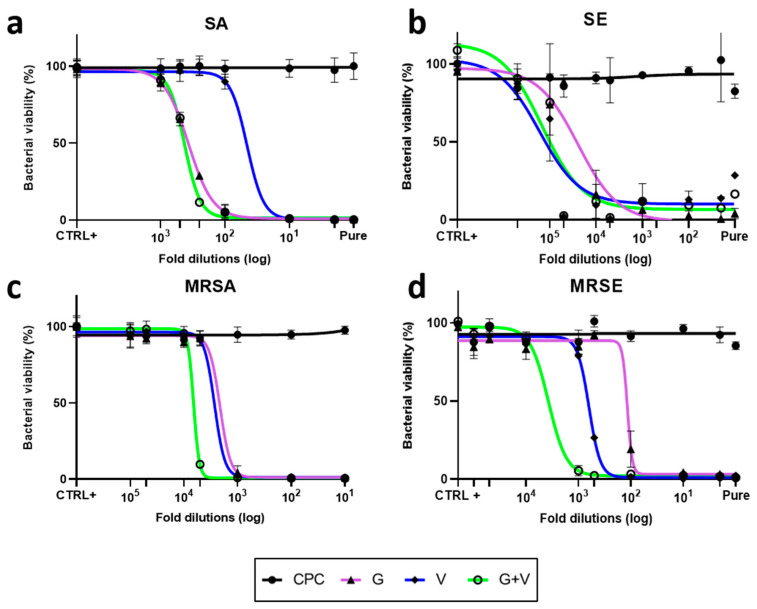
Antibacterial activity of CPC doped with gentamicin (G) and/or vancomycin (V) in liquid cultures. Bacterial viability of SA (**a**), SE (**b**), MRSA (**c**), and MRSE (**d**) after 4 h exposure to serial dilutions of cement eluates in LB, measured by fluorescence. CPC represents the reference group with unloaded pellets and the positive control is LB alone.

As shown in Figure 5a, vancomycin exhibited an IC_50_ approximately ten times lower than gentamicin. Interestingly, the combination of both antibiotics was as effective as gentamicin alone. For SE (Figure 5b), all three conditions—gentamicin, vancomycin, and their combination—produced comparable inhibitory effects. Notably, the antibiotic combination was more effective than either antibiotic alone against MRSA and MRSE (Figure 5c,d). A similar trend was observed for PA (Figure S1c).

IC_50_ values derived from liquid culture assays were consistent with those from agar diffusion tests (Table 1 and Table S1).

### 3.6. Effect on Bacterial Adhesion

**Figure 6:**
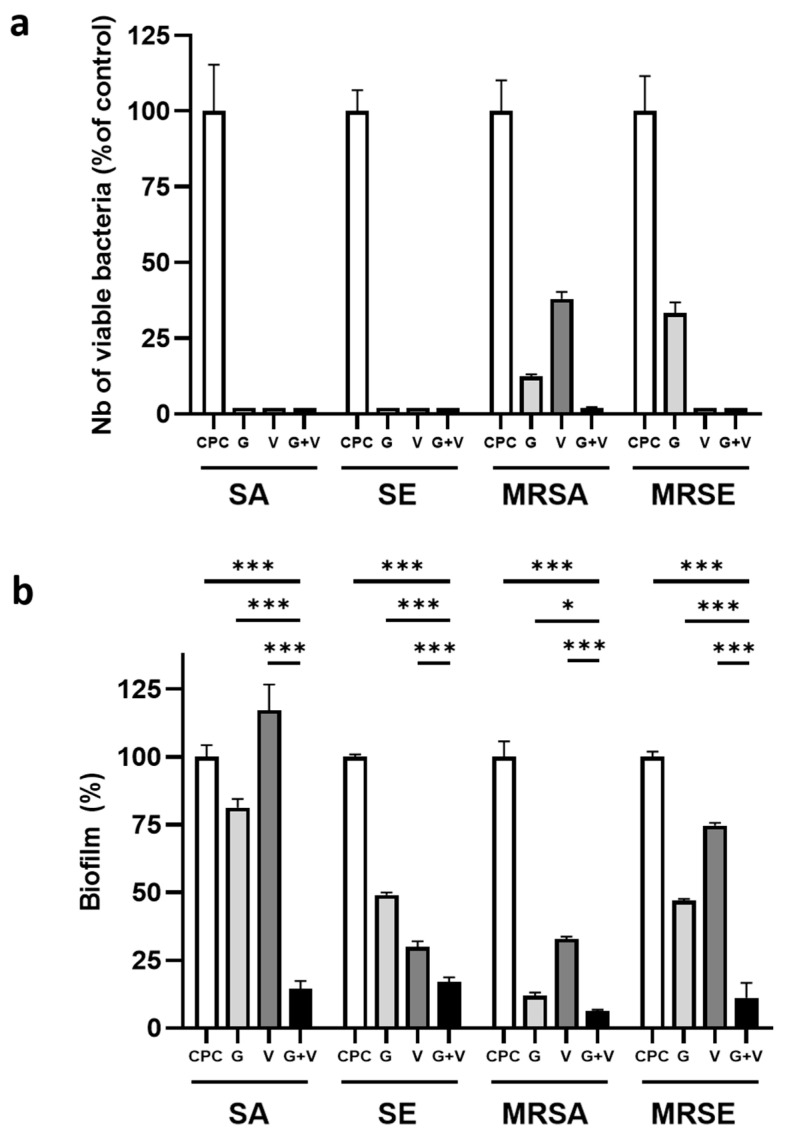
Anti-biofilm activity of CPC doped with gentamicin and/or vancomycin in microfluidic system. (**a**) Quantification of viable bacteria adhering to CPC pellets after 48 h exposure in a BioFlux200^TM^ system (0.2 dyne/cm^2^) at 37 °C for the different strains. Data are presented in % CPC for each strain. Each condition was measured on 4 pellets for each bacterial strain and is expressed as mean ± standard deviation (SD); (**b**) Percentage of channel area covered by biofilm after 48 h exposure in the microfluidic system at a flow rate of 0.2 dyne/cm^2^ and 37 °C. Data are presented in % CPC for each strain. Each condition was tested in triplicates and expressed as mean ± standard deviation (SD). Statistical significance: * *p* < 0.05; *** *p* < 0.001.

Given the clinical importance of biofilm-associated infections, we next evaluated the impact of antibiotic-loaded cements on bacterial adhesion, a critical early step in biofilm formation. As shown in Figure 6a, all strains adhered readily to the control CPC surface. The incorporation of either gentamicin or vancomycin completely inhibited adhesion of SA and SE. For MRSA, gentamicin strongly reduced adhesion by 87%, while vancomycin achieved a 62% reduction. Against MRSE, CPC + G reduced adhesion by 66.7%. Interestingly, the dual-antibiotic formulation fully inhibited adhesion of all Gram-positive strains. For the Gram-negative PA strain, vancomycin reduced adhesion by 73%, while gentamicin was even more effective, achieving an 88% reduction. The combination of both antibiotics nearly eradicated adhesion, with a 94% reduction (Figure S2f).

### 3.7. Biofilm Formation

Biofilm formation was further investigated using eluates from the different cement formulations. CPC + G was effective against all strains, reducing significantly biofilm formation to values comprised between 82.3% for SA and 11.9% MRSA (Figure 6b). Globally, CPC + V had a more reduced effect on biofilm formation than CPC + G on SE (30%), MRSA (32.9%), and MRSE (74.7%), and it was not effective at all against SA. This trend was also observed in the PA strain. CPC + G and CPC + V reduced biofilm formation to 8.5% and 21%, respectively (Figure S1e).

Importantly, the dual-antibiotic combination consistently outperformed single-antibiotic treatment, reducing biofilm formation to as low as 5% for PA and never exceeding 17.2% (for SE). Intermediate values were recorded for MRSA (6.4%), MRSE (11.2%), and SA (14.6%). All differences between the combination and single-antibiotic treatments were statistically significant (*p* < 0.001), except for PA, where gentamicin and the combination showed similar efficacy.

Given the polymicrobial nature of osteomyelitis, biofilm formation was also assessed in mixed-strain cultures. Results mirrored those from monocultures (Figure S2), confirming the superior efficacy of the dual-antibiotic-loaded cement.

Altogether, these findings demonstrate that incorporating both gentamicin and vancomycin into CPC significantly reduces bacterial adhesion and biofilm formation on cement surfaces.

## 4. Discussion

In this study, we developed a novel CPC formulation based on the clinically approved Quickset injectable bone graft co-loaded with two antibiotics: gentamicin and vancomycin. These antibiotics were selected due to their broad-spectrum activity and widespread clinical use against the primary pathogens responsible for osteomyelitis.

Both antibiotics were incorporated as powders into the solid phase of the cement without altering its physiochemical properties. Indeed, injectability remained constant across all formulations, ensuring ease of use in surgical settings. The most notable change was an increase in setting time. However, even with the addition of both antibiotics, it remained under 20 min, within the acceptable range for surgical application [48,49,50]. The prolonged setting time may be attributed to the interference of the antibiotics with the dissolution/precipitation reaction of calcium-deficient apatite, thereby slowing down the setting of the cement.

The antibiotics disrupt the cement dissolution/precipitation reaction by chelating with the calcium ions released during the alpha-TCP dissolution. However, the addition of antibiotics only has a visible impact on alpha-TCP dissolution when they are added together, as reflected by the alpha-TCP peaks in the CPC G + V diffractogram. The antibiotics also act as nucleating agents, competing with mineral precipitation on the alpha-TCP particles [51,52]. Similar findings have been reported by Bohner et al. [53] and Canal et al. [54], who observed that antibiotics such as gentamicin and doxycycline act as chelating agents, binding calcium ions released during the dissolution of alpha-TCP and thereby slowing the reaction by almost doubling the setting time. The presence of residual alpha-TCP is not problematic, as this phase has been reported to be biocompatible [55] and continues to convert into calcium-deficient apatite post-implantation.

Although longer setting times are often associated with a reduction in cement cohesion and consequently an increased risk of delamination, our results showed no evidence of delamination. Moreover, porosity and macrostructure, as observed by SEM, remained unaffected.

Decreased peak intensity in XRD indicates that particle size is reduced by the addition of antibiotics, confirming their ability to act as nucleating agents and promote more homogeneous nucleation, not just on alpha-TCP particles [53]. This should imply a reduction in both porosity and pore size, which cannot be estimated using the first porosity measurement we used.

To confirm this hypothesis, the mercury porosity experiment was carried out to study the pore size distribution with the addition of antibiotics (Table S2). Decreases in both particle and pore size can lead to a reduction in the mechanical properties of the cement. The results for mechanical strength under wet conditions corroborate this hypothesis, showing a greater reduction in the cement’s mechanical properties when porosity is lower (CPC + G and CPC G + V). The difference in results between wet and dry conditions can be explained by the drying phase allowing crystals to form, resulting in greater mechanical strength due to the presence of CDA crystals.

Most cements must comply with American Society for Testing and Materials (ASTM) standards. However, unlike PMMA cements, there is no ASTM standard describing the behaviour of apatitic cements in terms of compressive or flexural strength. In the past, the ASTM standard F451 [56] was used as a reference, but this is no longer the case. As far as flexural strength is concerned, to our knowledge, there are no reference standards for cements used in bone filling. This seems logical, given that the pathologies treated are non-critical defects and that cements are not likely to be subjected to such mechanical stress.

However, the mechanical resistance values obtained are comparable to those described for calcium sulphates mixed with an antibiotic already on the market [57,58]. In addition, low compressive strength values are not a problem, as the defects filled are non-critical and, after implantation, patients are immobilised for 2 to 6 weeks, depending on the area implanted, in order to limit mechanical stress on the bone but also on the implant.

Our data showed that the antibiotic release profile demonstrated a characteristic ‘burst’ effect, with high concentrations released within the first 24 h. The release follows a hindered Fickian diffusion which corresponds to the Korsmeyer–Peppas mathematical model used by Fosca et al. [59] with 0 < *n* < 0.45, meaning that the antibiotic release is mainly controlled by the diffusion through the cement matrix (Table S3). This is consistent with previous reports by Vorndran et al. [22] regarding a powder/liquid cement loaded with 1.24% (*w*/*w*) gentamicin or vancomycin and by Su et al. [20] regarding a biphasic α-tri-calcium phosphate/hydroxyapatite cement containing 4% (*w*/*w*) of gentamicin. Such a burst release will ensure a high local antibiotic concentration in the first few hours, leading to an optimal eradication of bacterial strains present at the infection site. Using the protocol established by Stravinskas et al. [43] that mimics the in vivo washing process, we showed that both antibiotics were released over 49 days at concentrations exceeding the MICs recommended by EUCAST (https://www.eucast.org/clinical_breakpoints, accessed on 28 May 2025). Sustained release above MIC levels is critical not only for eradicating pathogens at the infection site but also for preventing the emergence of antibiotic resistance.

Importantly, the high local antibiotic concentrations did not induce cytotoxic effects on human osteoblasts, meeting the ISO 10993-5 cytocompatibility standard [60]. These results are consistent with previous studies demonstrating the biocompatibility of CPCs loaded with gentamicin [61] or vancomycin [62]. While most studies reported in the literature [18,30,63] assess the antibacterial activity of antibiotic-loaded carriers using agar diffusion assays alone, we extended these findings by evaluating the efficacy of antibiotics release on planktonic cultures, which better mimic physiological conditions. Our results confirmed strong antibacterial activity against both methicillin-sensitive and methicillin-resistant Gram-positive strains. Notably, the dual-antibiotic combination was more effective than either antibiotic alone against MRSA and MRSE, suggesting a potential synergistic effect between the two antibiotics. This dual-antibiotic strategy may offer a rapid and localised antibacterial effect, overcoming the limitations of systemic antibiotic delivery into infected tissues, particularly the poorly vascularised bone [5,6].

Bacterial adhesion is the first step in biofilm formation, a key factor in chronic osteomyelitis, as described by Schilcher and Horswill [64]. Previous studies using dual-antibiotic-loaded cements have shown antibiofilm effects, though typically against a limited number of strains. For example, Jiang et al. [30] demonstrated reduced adhesion and biofilm formation of SA and PA using a calcium sulphate cement (Stimulan G + V™) loaded with gentamicin (1.2%) and vancomycin (5%). Similarly, Cara et al. [65] showed a strong reduction in biofilm formation by PA using a PMMA-based cement (COPAL G + V™) loaded with 0.5 g gentamicin and 2 g vancomycin.

In our study, the combination of gentamicin and vancomycin completely inhibited the adhesion of all Gram-positive strains tested to the CPC surface and significantly reduced the adhesion of PA. Furthermore, biofilm formation was markedly suppressed across all tested strains, including MRSE, where each antibiotic alone showed limited efficacy in preventing biofilm formation. These effects were also observed in mixed-strain cultures, reinforcing the clinical relevance of this approach. To our knowledge, this is the first report demonstrating both anti-adhesive and antibiofilm effects of a dual-antibiotic-loaded CPC material. Nevertheless, this study has limitations. In particular, in vivo validation is needed to assess cement degradation over time, local inflammatory responses, and potential systemic toxicity associated with high local concentrations of the applied antibiotics, especially given the nephrotoxic potential of vancomycin. Future studies should evaluate the in vivo efficacy of this cement in an infectious model and investigate its long-term safety profile, including potential impacts on renal function. Revalidation by the Food and Drug Administration (FDA) would not be necessary. The aim is not to add antibiotics to the kit ourselves, but rather to provide recommendations in the GQS leaflet for surgeons who wish to prevent infection by adding the two tested antibiotics to GQS cements. The instructions will therefore specify the addition of antibiotics in certain proportions and the resulting changes to the cement’s intrinsic properties.

## 5. Conclusions

This study aimed to develop a calcium phosphate-based cement co-loaded with two antibiotics (gentamicin and vancomycin) to achieve broad-spectrum antibacterial activity while preserving the intrinsic properties of the base material. The modified Graftys ^®^Quickset cement retained its key physicochemical properties, including phase composition and macrostructure. Although the setting time was extended when loaded with two antibiotics, it remained within the clinically acceptable threshold of 20 min. The dual-antibiotic-loaded cement demonstrated potent antibacterial effects against a variety of strains, including methicillin-sensitive and methicillin-resistant Gram-positive bacteria, a Gram-negative bacillus, and polymicrobial cultures. These effects were observed in both direct contact assays and as planktonic cultures, reflecting clinically relevant infection scenarios. Notably, the combination of gentamicin and vancomycin exhibited synergistic effects against strains with limited sensitivity to either antibiotic alone, thereby enhancing overall antibacterial efficacy. Additionally, the initial burst release of antibiotics did not induce cytotoxicity in human osteoblasts, ensuring compliance with the ISO 10993-5 cytocompatibility standards. Altogether, these findings suggest that this novel dual-antibiotic-loaded CPC represents a promising solution comparable to rapidly resorbing CS-based cements but with two antibiotics. It offers localised infection control, making it a valuable candidate for orthopaedic applications in the management of osteomyelitis.

## Figures and Tables

**Table 1:** IC_50_ values in µg/mL calculated using GraphPad Prism for each bacterial strain.

Strains	IC_50_ G (µg/mL)	IC_50_ V (µg/mL)	IC_50_ G + V (µg/mL)
SA	2.6	96.2	2.0
MRSA	0.4	1.4	0.1
SE	0.4	0.2	0.2
MRSE	8.3	5.4	0.9

## Data Availability

The data supporting the findings of this study are available within the article and its Supplementary Materials. Additional raw data are available from the corresponding authors upon reasonable request.

## Supplementary Materials

The following supporting information can be downloaded at: https://www.mdpi.com/article/10.3390/jfb16090320/s1, Supplementary Figures S1–S3 and Tables S1–S3 provide detailed data on the antibacterial and anti-biofilm activity, IC50 values, mechanical strength, porosity, and release kinetics of CPCs doped with gentamicin and/or vancomycin. The supplementary materials include Figure S1, which shows the antibacterial activity of CPC doped with gentamicin and/or vancomycin against *Pseudomonas aeruginosa*, including inhibition zones, bacterial viability, IC50 determination, bacterial adhesion under flow, and biofilm coverage; Table S1 summarizes the corresponding IC50 values. Figure S2 presents the anti-biofilm activity of antibiotic-loaded CPC against mixed MRSA–*P. aeruginosa* cultures, reporting bacterial adhesion and biofilm coverage under flow. Figure S3 evaluates the effect of gentamicin and/or vancomycin addition on cement strength, with flexural and compressive testing, while Table S2 details porosity and pore size measurements of the different CPC formulations. Finally, Table S3 provides R^2^ values for mathematical models fitting the release kinetics of gentamicin and vancomycin from CPC.
