# Supplementary material for: Antibiofilm Activity of a Novel Calcium Phosphate Cement Doped with Two Antibiotics
Source: J Funct Biomater. 2025 Aug 31;16(9):320. doi: 10.3390/jfb16090320 (PMC12470291; doi:10.3390/jfb16090320)
Supplement: Supplementary file 1 [file jfb-16-00320-s001.zip › jfb-3748075-supplementary.pdf]

Supplementary Materials

Antibiofilm activity of a novel Calcium-Phosphate Cement doped with two antibiotics

Eneko Elezgaray[1,2,3], Cassandra Pouget[4], Fanny Salmeron[2], Catherine Flacard[3],

Jean-Philippe Lavigne[4], Vincent Cavaillès[2*], Mikhael Bechelany[1*]

[1] Institut Européen des Membranes, IEM, UMR 5635, University of Montpellier, ENSCM, Centre national de la recherche scientifique (CNRS), Place Eugène Bataillon, 34095 Montpellier.

[2] IRCM, Institut de Recherche en Cancérologie de Montpellier, INSERM U1194, Université Montpellier, Montpellier F-34298, France

[3] NORAKER SAS, 60 Av. Rockefeller, 69008 Lyon, France

[4] VBIC, INSERM U1047, Univ Montpellier, Service de Microbiologie et Hygiène Hospitalière, CHU Nîmes, Nîmes, France.

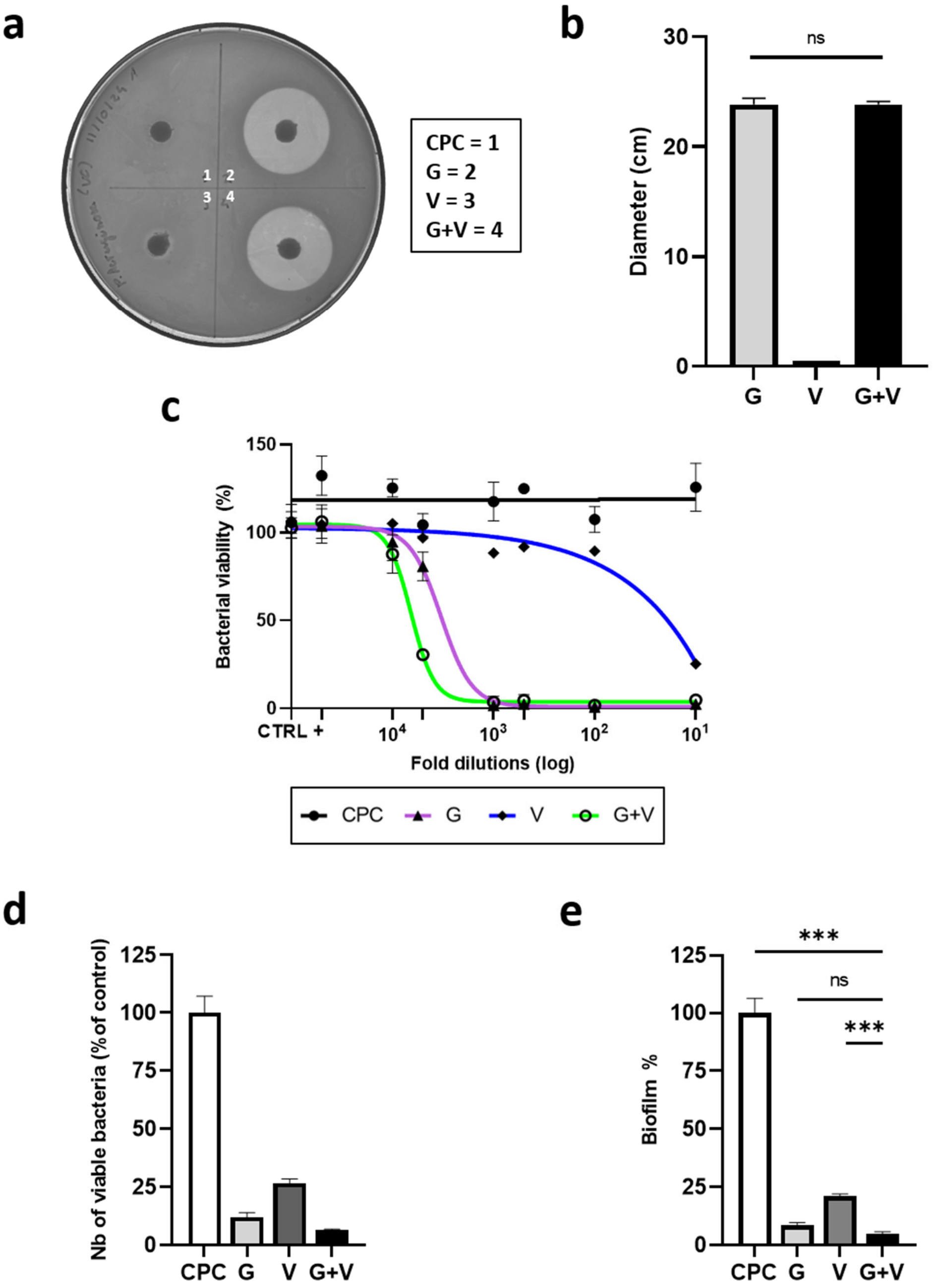

Figure S1 - Antibacterial activity of CPC doped with gentamicin and/or vancomycin against *Pseudomonas aeruginosa*

(a) Representative images of inhibition zones of the antibiotic loaded CPCs ((1) CPC, (2) CPC+G, (3) CPC+V, (4) CPC+G+V) after 24h incubation at 37°C; (b) Quantification of inhibition zone diameters. Experiments were performed in triplicate, and data expressed as mean ± standard deviation (SD); (c) Bacterial viability measured by fluorescence after 4h exposure to eluates in LB; (d) Quantification of viable PA adhering to CPC pellets after 48h in microfluidic flow rate of 0.2 dyne/cm² and 37°C. Results are presented in %CPC for each strain. Each condition was measured on 4 pellets for each bacterial strain and is expressed as mean ± standard deviation (SD); (e) Percentage of biofilm coverage in channels after 48h exposure to cement eluates at a flow rate of 0.2 dyne/cm² and 37°C. Results are presented in %CPC for each strain. Each condition was measured in triplicates for each bacterial strain and is expressed as mean ± SD. Statistical significance: ns $p>0.32$; ***$p<0.001$.

Table S1- IC_{50} values in µg/ml calculated using GraphPad Prism for *Pseudomonas aeruginosa*.

Strain	IC_{50} G (µg/ml)	IC_{50} V (µg/ml)	IC_{50} G+V (µg/ml)
**PA**	0.3	148.3	0.1

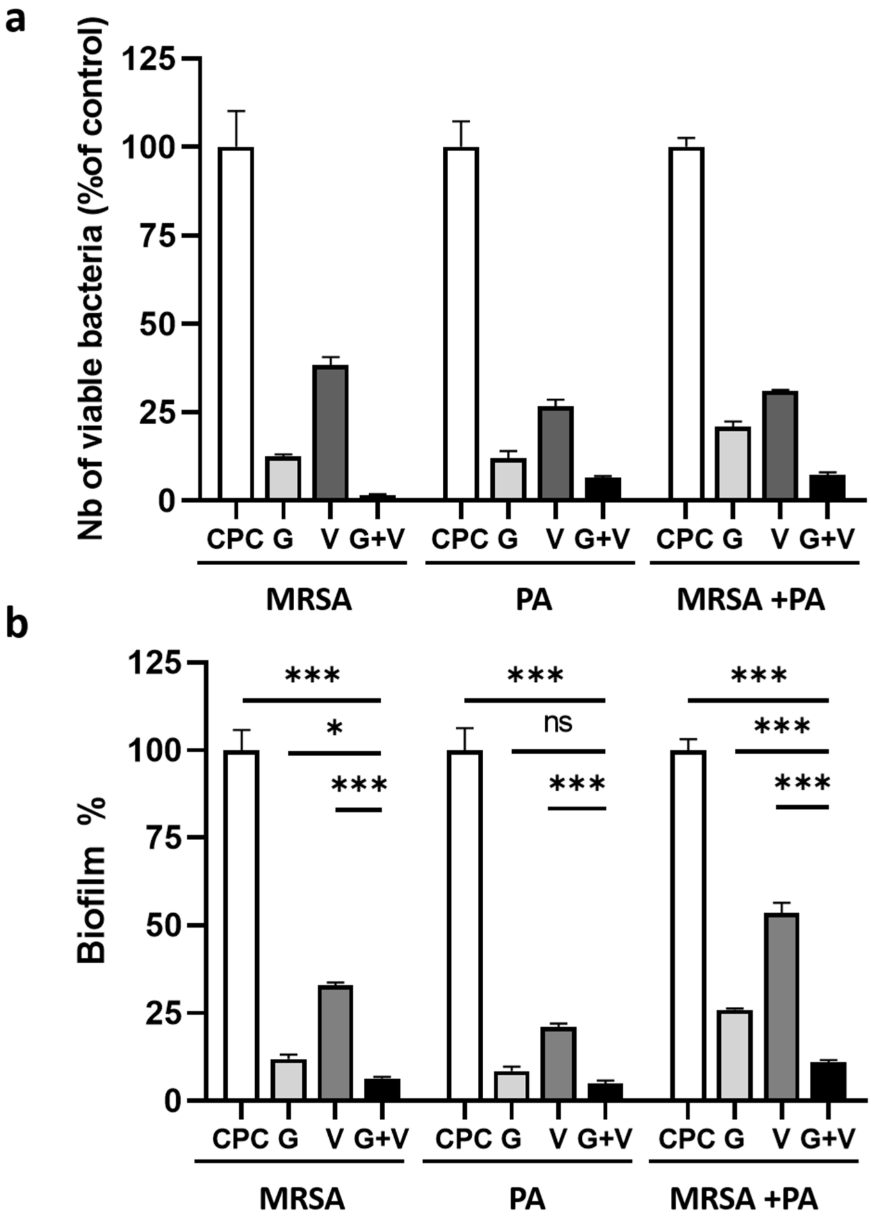

Figure S2 – Anti-biofilm activity of CPC doped with gentamicin and/or vancomycin against a mixed MRSA-*P. aeruginosa* culture

(a) Quantification of viable bacteria adhering to CPC pellets after 48h in microfluidic flow rate of 0.2 dyne/cm² and 37°C for different strains. Results are presented in %CPC for each strain**.** Each condition was measured on 4 pellets for each bacterial strain and is expressed as the mean ± standard deviation (SD); (b) Percentage of biofilm coverage in channels after 48h exposure to cement eluates at a flow rate of 0.2 dyne/cm² and 37°C. Results are presented in % CPC for each strain**.** Each condition was measured in triplicates for each bacterial strain and is expressed as mean of the values ± SD. Statistical significance: ns $p>0.32$; $*p<0.05$; $***p<0.001$.

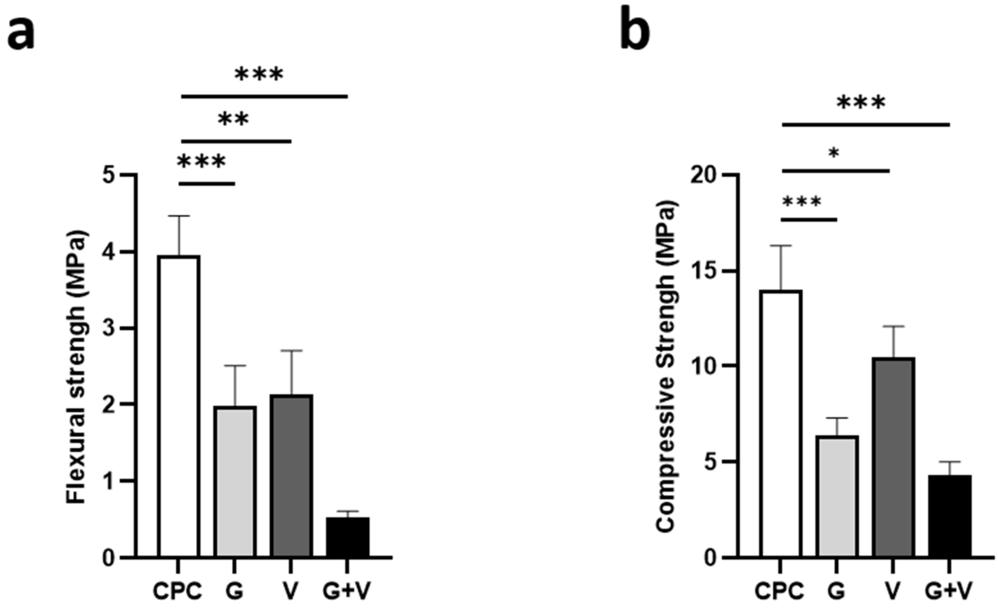

Figure S3 – Effect of the addition of gentamicin and/or vancomycin on cement strength.

(a) Flexural and (b) compressive strength measured on 6 samples per conditions. CPC refers to the unloaded control cement. Data are presented as mean ± standard deviation (SD). Statistical significance: ns p>0.32; *p<0.05; **p<0.01; ***p<0.001.

Table S2- Porosity (%) and average pore diameter (µm) measured on cylindrical samples (n=1) of the different conditions studied by mercury porosity.

Conditions	Porosity (%)	Average pore diameter (µm)
**CPC**	47.3	0.0246
**G**	45.8	0.0167
**V**	47.3	0.0224
**G+V**	43.6	0.0167

Table S3- R^2 coefficient values calculated from the released curves of gentamicin and vancomycin from CPC G+V for different mathematical models.

	R^2	
	**G in G+V**	**V in G+V**
**1 st order**	0.8211	0.9047
**2 nd order**	0.8496	0.9635
**Korsmeyer peppas**	0.9751	0.9886
